# Not Enough Fat: Mouse Models of Inherited Lipodystrophy

**DOI:** 10.3389/fendo.2022.785819

**Published:** 2022-02-18

**Authors:** Soazig Le Lay, Jocelyne Magré, Xavier Prieur

**Affiliations:** ^1^ Nantes Université, CNRS, INSERM, l’institut du thorax, Nantes, France; ^2^ Univ Angers, SFR ICAT, Angers, France

**Keywords:** adipocyte, lipodystrophy, insulin resistance, cardiometabolic abnormalities, diabetes

## Abstract

Lipodystrophies belong to the heterogenous group of syndromes in which the primary defect is a generalized or partial absence of adipose tissue, which may be congenital or acquired in origin. Lipodystrophy should be considered in patients manifesting the combination of insulin resistance (with or without overt diabetes), dyslipidemia and fatty liver. Lipodystrophies are classified according to the etiology of the disease (genetic or acquired) and to the anatomical distribution of adipose tissue (generalized or partial). The mechanism of adipose tissue loss is specific to each syndrome, depending on the biological function of the mutated gene. Mice models, together with cellular studies have permitted clarification of the mechanisms by which human mutations deeply compromise adipocyte homeostasis. In addition, rodent models have proven to be crucial in deciphering the cardiometabolic consequences of the lack of adipose tissue such as NAFLD, muscle insulin resistance and cardiomyopathy. More precisely, tissue-specific transgenic and knockout mice have brought new tools to distinguish phenotypic traits that are the consequences of lipodystrophy from those that are cell-autonomous. In this review, we discuss the mice models of lipodystrophy including those of inherited human syndromes of generalized and partial lipodystrophy. We present how these models have demonstrated the central role of white adipose tissue in energetic homeostasis in general, including insulin sensitivity and lipid handling in particular. We underscore the differences reported with the human phenotype and discuss the limit of rodent models in recapitulating adipose tissue primary default. Finally, we present how these mice models have highlighted the function of the causative-genes and brought new insights into the pathophysiology of the cardiometabolic complications associated with lipodystrophy.

## Introduction

Inherited lipodystrophies belong to the heterogeneous group of syndromes characterized by a lack of adipose tissue (AT) associated with insulin resistance, hypertriglyceridemia, and non-alcoholic fatty liver disease (NAFLD) (1, 2). According to the severity and the anatomical distribution of AT, lipodystrophy could be generalized or partial (3).

Generalized lipodystrophy or Berardinelli–Seip congenital lipodystrophy (BSCL) is characterized by an almost complete lack of AT from birth or early infancy. Severe insulin resistance (assessed by the presence of acanthosis nigricans) progresses to overt diabetes during the teenage years or later. BSCL is a rare heterogeneous recessively inherited disorder (4).

Partial lipodystrophies are characterized by a stereotypic pattern of AT loss affecting the limbs and normal or excess fat on the face and the neck (3). The reason for the fat depot phenotypical differences remains unknown. Metabolic features range from asymptomatic impaired glucose tolerance with mild dyslipidemia to severe insulin resistance, diabetes and NAFLD (5). The familial partial lipodystrophy (FPLD) syndromes are usually transmitted according to an autosomal dominant mode of inheritance (3).

**Figure 1:**
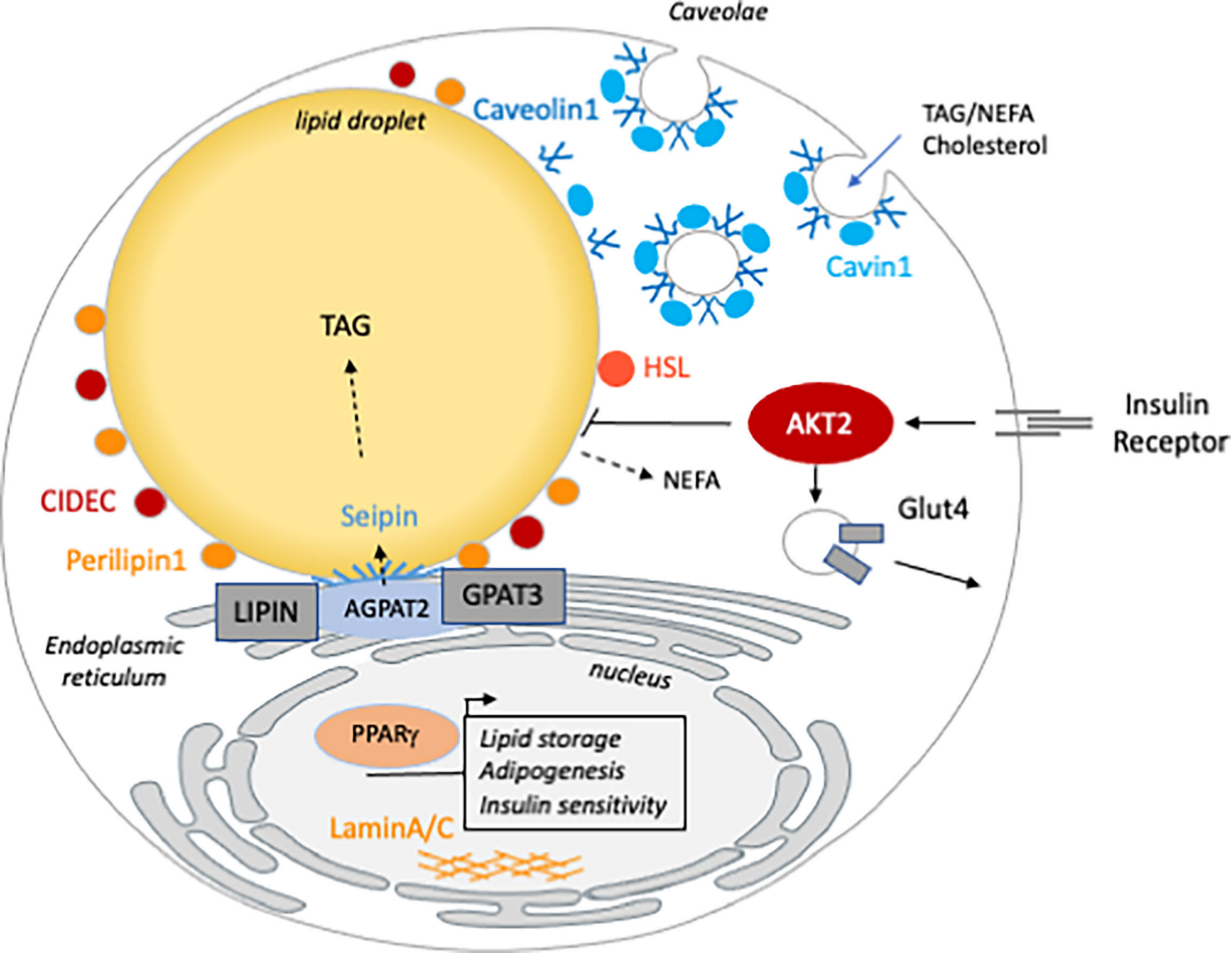
The cellular function of the genes mutated in inherited lipodystrophy. Genes involved in generalized (blue) are involved in TG synthesis (AGPAT2), LD (BSCL2/seipin) or caveolae (Caveolin1 and Cavin1) homeostasis. Partial lipodystrophy causative genes (orange) are involved in different functions of the mature adipocyte. CIDEC, PLIN1 and HSL are LD associated and/or involved in lipolysis regulation. AKT2 and PPARG are both involved in insulin sensitivity. Mutations in LMNA, the gene encoding the nucleophilic lamins A/C, are the most frequent cause of FPLD but how they lead to lipodystrophy remains poorly understood.

The mechanism of AT loss is specific to each lipodystrophic disorder, depending on the biological function of the mutated gene (6) (**Figure 1**). Lipodystrophies are rare conditions and clinical studies are difficult to conduct. In BSCL, the absence of AT may occur at birth or in infancy and is the first sign of the condition. Therefore, historically, it has been difficult to know whether the lack of AT was related to a developmental defect or to a rapid and massive loss of mature adipocytes. Mice models, together with cellular studies allowed the identification of the mechanisms by which human mutations can deeply compromise AT homeostasis. Similarly, rodent models appeared to be crucial to decipher the cardiometabolic consequences of the lack of AT such as NAFLD, muscle insulin resistance and cardiomyopathy. More precisely, tissue-specific genetically modified mice brought new tools to distinguish phenotypical traits that are the consequences of lipodystrophy or that can be attributed to cell autonomous mechanisms.

In this review, we discuss how mouse models of inherited lipodystrophy have demonstrated the central role of white AT (WAT) in energetic homeostasis in general, including insulin sensitivity and lipid handling in particular. Then, we present how the different mice carrying gene deletions involved in generalized and partial lipodystrophies highlight the function of these genes and brought new insights into the pathophysiology of the cardiometabolic complications associated with these pathologies.

## Life With No Fat: Mice Models of Generalized Lipodystrophy

### No Fat, No Good

In 1993, Spiegelman’s group have tested the effect of limiting AT expansion on metabolic health in the context of obesity (7). To this end, they have generated mice expressing low levels of a diphtheria toxin under the adipocyte-specific aP2 promoter. Whereas adipose-specific expression of the diphtheria toxin has no effect in lean mice, it resulted in a strong reduction of adiposity and resistance to weight gain in obese mice. Importantly, the mice displayed severe hypertriglyceridemia and liver steatosis demonstrating that impairing AT expansion has a deleterious effect on metabolic health.

A few years later, three mouse models of generalized lipodystrophy have been generated by modifying the expression of key transcription factors involved in adipogenesis: the adipocyte-specific over-expression of the nuclear mature form of SREBP1c (aP2-nSREBP1c) (8), the adipocyte specific expression of a dominant negative protein that binds to C/EBPα (A-ZIP/F mice) (9) and the suppression of C/EBPα expression in the AT (10). All three mice presented with a nearly complete lack of AT, diabetes and hepatic steatosis, displaying therefore all the characteristics of BSCL. Importantly, surgical transplantation of AT in A-ZIP/F mice (11), as well as leptin supplementation of aP2-nSREBP1c mice (12), strongly improved the insulin-resistance and liver steatosis, pointing out that the lack of AT is central to the development of the metabolic complications associated with lipodystrophy. Those mice could be considered as the first models of generalized lipodystrophy even though the genetic cause was not the one identified in humans. Later on, the identification of the genes responsible for BSCL in humans led to the development of new rodent models of generalized lipodystrophy.

### The Triglyceride Synthesis Key Player AGPAT2 Controls Adipocyte Maintenance

1-acyl-sn-glycerol-3-phosphate acyltransferases (AGPAT) are key enzymes involved in lipid synthesis. The isoform AGPAT2, highly expressed in the AT, catalyses the acylation of lyso-phospatidic acid (LPA) to produce phosphatidic acid (PA) that will subsequently enter the triglyceride (TG) or phospholipid synthesis. In humans, biallelic AGPAT2 mutations cause BSCL1 (13). Total AGPAT2 KO mice present with virtually no white nor brown AT (14). The AGPAT2 deficient mice are hyperglycemic, hyperinsulinemic, hypoleptinemic, insulin-resistant and display liver steatosis. Indirect calorimetry studies revealed that these mice have a constant respiratory quotient along the day suggesting that they are metabolically inflexible and unable to switch from one substrate to another according to the nutritional status (14). Interestingly, AGPAT2 re-expression in the liver of total KO mice did not rescue the massive liver steatosis (15) whereas leptin replacement did, as well as it improved glucose homeostasis (16). This reinforces the central role of leptin deficiency in the pathophysiology of BSCL. This strongly suggests that lipodystrophy was the cause of the metabolic disorders described in AGPAT2 KO mice. Therefore, several studies intended to describe AGPAT2 function in AT.

Cellular studies have supported that AGPAT2 is necessary for adipocyte differentiation, suggesting that the absence of WAT in AGPAT2 KO was the result of an altered adipogenesis (17, 18). Surprisingly, the characterization of the AT in foetuses, at birth and in the first week of life, revealed that the AGPAT2 KO new-borns have normal AT that is rapidly lost. Another study confirmed that WAT depots are normal until AGPAT2 KO mice were 1-week-old but completely absent in aged mice, supporting that AGPAT2 is required for mature adipocytes maintenance (19). Indeed, rather than a developmental issue, AGPAT2 deficiency induces autophagy, inflammation and massive cell death (20). Importantly, the apoptosis induction was associated with a strong accumulation of PA, an increase in phosphatidylcholine (PC) and a decrease in phosphatidylserine (PS), phosphatidylinositol (PI), and phosphatidylglycerol (PG). As PC increases and PE remains unchanged, PE/PC (20) ratio likely drops thereby inducing cellular stress and cell death as previously demonstrated (21). Of note, AGPAT2 KO adipocytes are devoid of caveolae (20), a subclass of membrane microdomains involved in adipocyte fat storage and whose deficiency is associated with lipodystrophy (see later).

Interestingly, Lipin-1, the enzyme that catalyses the transformation of PA into diacylglycerol (DAG) has been involved in lipodystrophy in mice only (22), not in human (23). Spontaneous loss-of-function mutations in the gene encoding Lipin-1 have been identified in the fatty liver dystrophic (FLD) mice, characterized by a massive liver steatosis in the pre-suckling period (24) and a severe lipodystrophy associated with glucose intolerance in adult animals (25). In mice, adipocyte restricted deletion of Lipin-1 strongly affects adipocyte TG synthesis, leads to PA accumulation, and induces lipodystrophy (26). Lipin-1 is both a co-regulator DNA binding factor and a PA-phosphatase enzyme involved in TG synthesis. Importantly, a unique mouse model has been generated with a truncated Lipin-1 lacking the lipid synthesis activity but retaining the DNA binding domain (27). Those mice display severe adipose tissue loss supporting that the TG synthesis activity of Lipin-1 is crucial for adipocyte maintenance. Taken together with the lessons from AGPAT2 deficiency, these findings demonstrate that the TG synthetic capacity of the adipocyte is a crucial determinant of adipocyte maintenance.

### BSCL2 Encodes the Mysterious Protein Seipin

Mutations in the gene *BSCL2* have been the first genetic explanation for generalized lipodystrophy in humans (28). *BSCL2* encodes seipin whose biological function remains poorly understood, especially in adipocytes. *In vitro* studies report that seipin is involved in lipid droplet (LD) homeostasis and in LD/ER (endoplasmic reticulum) contact sites [for review (29)]. *BSCL2* transcripts are highly expressed in brain, AT and testis. Different animal models have been generated to better describe the pathophysiology of BSCL2. Initially, three total and constitutive seipin deficient (SKO) mice have been studied and showed similar characteristics (30–32). 8 to 12-week-old mice display a near-complete lack of WAT (90% reduction), insulin-resistance and hepatic steatosis. A loss of 60 to 50% of brown AT (BAT) mass is also observed. Unexpectedly, SKO mice are hypotriglyceridemic, in contrast to human BSCL2 patients who display elevated TG levels. One study proposed that this low TG levels might be due to an increase in TG-rich lipoprotein uptake in the liver of SKO mice (30). Of note, BSCL2 deficient rat are hypertriglyceridemic suggesting that rat could be a better model to study lipoproteins in the context of lipodystrophy (33). In the absence of energy storage, these mice are intolerant to fasting and exhibit metabolic inflexibility (31, 34). SKO mice show a decrease in TG and an increase in glycogen in skeletal muscles (35). Seipin deficiency induces renal dysfunction associated with elevated glycation and TG levels in SKO glomerular area (36). Since AT transplantation and leptin replacement improve the renal function, the kidney phenotype is likely a consequence of the lipodystrophy and not a cell autonomous function of seipin. A recent study reported a pancreatic phenotype characterized by a beta-cell hypertrophy and an alteration of the insulin secretion profile in response to a glucose bolus (37). Intriguingly, this study showed that the heterozygous deletion of seipin is sufficient to lead to beta-cell dysfunction whereas it does not alter the AT mass, suggesting a cell autonomous action of seipin in beta-cells. Further studies with a pancreatic-specific deletion of seipin are needed to confirm this hypothesis.

Three studies have also reported the rapid development of diabetic cardiomyopathy characterized by left ventricular hypertrophy, cardiac insulin resistance and diastolic dysfunction (38, 39). We have shown that in SKO mice, cardiac dysfunction is associated with hyperglycemia, cardiac glucose overload and more precisely with a chronic activation of the hexosamine biosynthetic pathway (HBP). Interestingly, SGLT2 inhibitor (dapagliflozin) treatment normalized the plasma glucose level, decreased the chronic activation of the HBP, and improved the cardiac phenotype of SKO mice (38). The second study proposed that cardiac dysfunction is related to chronic activation of FA oxidation in SKO heart as a consequence of uncontrolled lipolysis. Indeed, inhibition of adipose tissue TG lipase (ATGL) ameliorates the lipodystrophic phenotype and consequently corrects cardiac dysfunction (39). The last report incriminates changes in the phosphorylation levels of the sarcomeric protein Titin (40). In this report, cardiac specific deletion of seipin did not lead to heart abnormalities suggesting that cardiomyopathy is a consequence of lipodystrophy and not an autonomous cardiac dysfunction (40).

In order to address the question of the central role of the adipocyte seipin deficiency and subsequent lipodystrophy in the pathophysiology of BSCL2, several genetic animal models have been created. First of all, BSCL2 re-expression specifically in the adipocytes, through the aP2 promoter, is sufficient to correct the SKO mice lipodystrophy, insulin resistance and liver steatosis (41). At the opposite, liver-specific seipin deficiency (42, 43) does not induce liver steatosis nor insulin resistance, discarding an autonomous role of seipin in the hepatocyte at the origin of the liver complications reported in BSCL2 patients. Adipocyte-specific seipin deletion, either under the aP2 promoter (44) or the AdipoQ promoter (45), leads to progressive lipodystrophy. Under the aP2 promoter, the lipodystrophy is associated with all the associated metabolic complications (insulin resistance, glucose intolerance and liver steatosis) (44). In the second model, the metabolic complications are severely marked only under high-fat diet (HFD). Regarding the origin of lipodystrophy in the BSCL2 phenotype, *in vitro* experiments support that seipin is crucial for normal adipogenesis (46). However, adipogenesis impairment cannot fully explain the SKO severe lipodystrophy, as in these mice, we reported a loss of WAT mass and a decrease in circulating adiponectin levels between 4 to 12 weeks of age (47). Consistently, inducible adipose-specific seipin deletion compromises adipocyte survival and results in elevated basal lipolysis, leading to progressive AT loss (48). Therefore, seipin might play a role in both, adipocyte differentiation and maintenance of the full mature adipocyte phenotype.

### The Unexpected Involvement of Caveolae

In humans, mutations in *CAV1* encoding the caveolae protein caveolin-1, lead to a near complete loss of subcutaneous and visceral AT, associated with insulin resistance and dyslipidemia, therefore referred as BSCL3 (49). BSCL4 is caused by loss-of-function mutations in *CAVIN1/PTRF* (Polymerase I and Transcript Release Factor) encoding a required protein for caveolae biogenesis which regulates the expression of caveolins (50–52).

Caveolin-1 is a key structural protein of caveolae, omega-shaped membranous invaginations, that, together with cavin adaptor proteins, decorated almost 30% of adipocyte plasma membrane (53). Although caveolin-1 and/or cavin-1 deficiency leads to complete loss of caveolae structures, mice are still fertile and viable (54–56). Despite normal AT depots at birth, caveolin-1 null mice display a progressive lipoatrophy aggravating with age, although developing with a slightly different time frame depending on the KO model considered, characterized by the loss of hypodermal fat layer and generalized reduction of all WAT depots, hypertriglyceridemia but very mild insulin resistance as soon as 3 months of age (54, 57). CAV1 KO mice are moreover resisting to the development of obesity when fed a HFD (54, 58). They also exhibit elevated triglycerides and reduced leptin and adiponectin and overt diabetes only developed in the context of prolonged HFD (59). Whereas the leanness of CAV1 KO mice has been shown to be independent of altered energy expenditure, food intake or intestinal absorption (54), their complex metabolic phenotype has been related to metabolic inflexibility and mitochondrial dysfunction (60).

The generation of an adipocyte-specific KO of caveolin-1 was unsuccessful since the efficient exosomal trafficking of caveolin-1 from neighbouring endothelial cells compensates the adipocyte caveolin-1 deficiency (61).

Cavin-1 invalidation reproduced typical BSCL phenotype with significant fat loss, histological abnormalities of AT including marked fibrosis, and a significant decrease in circulating levels of adiponectin and leptin. From a metabolic point of view, the mice also show glucose intolerance, hepatic and muscular insulin resistance and hypertriglyceridemia (55, 62). The similarities of the lipodystrophic phenotypes displayed by CAV1 and CAVIN1 KO mice tend to incriminate the absence of caveolae structures in the development of metabolic alterations. Nonetheless, we can exclude that those cellular mechanisms regulated by nuclear and/or cytosolic cavin-1 also participate to this metabolic phenotype, a speculation that would require further investigations. Overall, distinguishing between the specific role attributed to individual caveolae-forming proteins and the ones linked to caveolae microdomains is still technically challenging, given the fact that they are intrinsically linked, emphasizing the need to explore alternative molecular models for a better understanding of their respective contribution (63).

Among the metabolic pathways impacted by caveolae disappearance, the localization and internalization of the insulin receptor within these membrane microdomains (59) early identified caveolin-1 as a positive regulator of the insulin signalling pathway. Moreover, as a lipid-binding protein (64), caveolin-1 is thought to participate to lipid trafficking, between plasma membrane and the LD (65, 66) and to modulate LD phospholipid and protein surface composition (67). Caveolin 1 deficiency alters fatty acid uptake (68, 69) but adipocyte cell surface caveolae might also be sites of local triglycerides synthesis (70). In adipocytes, we further demonstrated a reciprocal regulation of membranous caveolae density and fat cell LD storage, highlighting caveolae as mediators of lipid-driven fat cell size adaptation and expandability (71).

Despite no major abnormalities in energy balance reported in CAV1 KO mice, the absence of caveolin-1 has been linked to reduced ability to change substrate use in response to feeding/fasting conditions, which has been referred to metabolic inflexibility (60). Since mature adipocytes are present in young mice and mouse embryonic fibroblasts from CAV1 KO mice differentiate into adipocytes, caveolin-1 is not *per se* required for the formation of new adipocytes (65, 72). Besides, lipoatrophy might result from exaggerated breakdown of WAT stored lipids, since both caveolin-1 and cavin-1 have been both shown to be critical in regulating lipase-induced lipid mobilization (72, 73). Nonetheless, the study of the lipolytic response of isolated adipocytes to beta-3 adrenergic agonists has revealed blunted rather exacerbated lipolysis in CAV1 KO mice (72). Altered response to pro-lipolytic signals in CAV1 null animals results in increased susceptibility to cell death, inflammation and fibrosis in WAT (74). We moreover revealed constitutive adipocyte activated autophagy in the absence of caveolin-1, that associates with altered protein turnover and accelerated protein degradation impairing many metabolic pathways (57). Cellular studies using cultured skin fibroblasts from patients also argue for a role of maladaptative autophagy in the absence of cavin-1 that contributes to insulin resistance (75). It remains so far unclear whether such degradative process directly impacts adipocyte cell death and/or renewal as a primary defect of lipodystrophy or whether it develops as an adaptive mechanism to counteract adipocyte dysfunction.

In summary, caveolin-1 deficient adipocytes have to face with a metabolic situation characterized by defective fatty acid mobilization and an altered insulin-dependent nutrient supply which both likely converge to induce autophagy. These metabolic stresses could have an additional impact on WAT remodelling processes and the development of an inflammatory state, that, altogether, may favour and contribute to the development of lipodystrophy.

### Generalised Lipodystrophy Is the Cause of Metabolic Complications

Collectively, BSCL mice models display severe metabolic complications. For BSCL2, adipocyte specific models and inducible deletion recapitulate most of the features of the total SKO mice. In addition, for AGPAT2 and BSCL2 KO mice, leptin replacement or AT surgical implantation strongly improved the metabolic phenotype including insulin resistance, liver steatosis and renal injuries. Recently, Kahn’s lab generated adipocyte-specific tamoxifen inducible insulin receptor deletion (Adipo-ind-IRKO) and demonstrated that 3 days after tamoxifen injection, mice display a massive adipocyte loss and severe insulin resistance (76). Importantly, leptin supplementation prevents the appearance of insulin resistance and liver steatosis, but did not improve adipose tissue mass and quality. Unexpectedly, 30 days after tamoxifen injection, the neo-differentiation of new adipocytes that express the insulin receptor induces a re-increase in AT mass and a correction of glucose homeostasis abnormalities. This report remarkably demonstrated that metabolic health is dynamically determined by AT and that among adipocyte properties, leptin secretion is central in its ability to control metabolic homeostasis.

## What Did We Learn From Partial Lipodystrophy Rodent Models?

### LMNA, the Most Common Cause of Familial Partial Lipodystrophy

The most frequent genetic cause of FPLD is mutations in *LMNA*, and among them, the R482Q mutation represents 80% of the cases. *LMNA* encodes the nucleophilic A-type lamins, lamin A and lamin C which are generated by different splicing within exon 10 of *LMNA*. Several mice models have been generated to understand how lamin A/C mutations cause FPLD. In LMNA-deficient mice, growth is decreased at two weeks-old and completely stopped at 4-weeks old, and the animals do not survive over 2 months-old. The origin of death is a severe and early muscular dystrophy that leads to posture abnormalities such as scoliosis and major heart dysfunction (77). Those mice also display complete AT loss without metabolic complications (78). Lipodystrophy is suspected to be secondary to the muscular dystrophy but the severity of the phenotype renders this model difficult to interpret. On the other hand, the adipocyte-specific LMNA deficiency leads to a reduction of WAT mass in male and female mice that was associated with mild hyperglycemia and hyperinsulinemia in females (79). Five weeks-HFD feeding leads to a more severe lipodystrophy and a more marked metabolic phenotype characterized by hyperglycemia, hyperinsulinemia, elevated TG and low adiponectin and leptin levels. Importantly, in adipocyte specific LMNA-deficient mice, the AT develops postnatally but progressively disappears from 4 weeks of age, suggesting that LMNA deficiency alters adipocyte maintenance. Consistently, *in vitro* studies support that LMNA deficiency does not impair adipogenesis of mesenchymal stem cells but accelerates lipolysis in differentiated adipocytes. This phenotype is quite consistent with that reported in FPLD patients carrying *LMNA* mutations.

Transgenic mice expressing human *LMNA* with the common R482Q mutation under the adipocyte specific aP2 promoter have been generated (80). Of note, in these mice, the transgene is present in the hemizygous state in addition to the two wild-type copies of the murine *Lmna* gene; while a knock-in introduction of the R482Q mutation would be closer to the situation of FPLD patients. Under HFD feeding, those mice develop lipodystrophy, abnormal AT histology, glucose intolerance, insulin resistance and liver steatosis. Adipose tissue loss consecutive to R482Q mutation introduction is similarly observed in all depots at the exception of the inguinal WAT which is not significantly impacted. The AT distribution and the metabolic phenotype of the R482Q mice resemble those reported in the adipocyte-specific LMNA-deficient mice. However, the *in vitro* data obtained with stromal cells isolated from the R482Q AT generate different results than those obtained with the mesenchymal cells from adipocyte-specific LMNA-deficient WAT. Indeed, *in vitro*, the R482Q mutation does not modify lipolysis rates but alters adipogenesis. Therefore, whereas the phenotype of mice with the R482Q mutation or adipocyte-specific LMNA deficiency are similar, the mechanism at the origin of lipodystrophy remains elusive.

### PPAR Gamma

PPARγ (Peroxisome proliferator-activated receptor gamma) is the master regulator of adipogenesis and total KO of PPARγ is lethal (81, 82). Adipocyte-specific deletion of PPARγ under the AdipoQ gene promoter leads to a nearly complete lack of AT, insulin resistance and massive liver steatosis, *i.e.* generalized lipodystrophy (83). In humans, a dominant-negative mutation in the ligand binding domain of PPARγ (P467L), is associated with severe insulin resistance, diabetes and hypertension (84), and further clinical characterization revealed a FPLD syndrome (85). Surprisingly, mice carrying the equivalent P465L mutation do not develop lipodystrophy nor insulin resistance under chow or HFD (86, 87) but display a change in fat distribution with an increase in subcutaneous fat pads and a decrease in gonadal WAT (86). This unexpected result suggests that in mice, P465L confers leanness rather than pathological lipodystrophy. In order to understand further the pathophysiological effect of the P465L mutation in mice, one group crossed these mice with the obese and hyperphagic leptin-deficient ob/ob mice model (88). The heterozygous P465L mutation on an ob/ob background leads to a reduction of WAT mass, the development of severe insulin resistance, lipid liver accumulation and alteration in post-prandial TG clearance (89). Of note, this was not the case after 16 weeks HFD feeding (89), suggesting that a stronger positive energy balance is needed to reveal the metabolic consequences of limited AT expansion due to P465L dominant-negative mutation of PPARγ. To conclude, whereas it is clear that adipocyte PPARγ deficiency leads to severe lipodystrophy, the FPLD phenotype due to P465L is not easy to recapitulate in mice and it appears that according to the body weight phenotype (lean, mild overweight or obese), this mutation will balance towards healthy leanness or lipoatrophic insulin resistant phenotype.

### Perilipin-1

Perilipin-1 is the most abundant LD-associated protein in mature adipocytes and its main biological function is to prevent basal lipolysis and to allow adrenergic stimulated TG hydrolysis (90). Heterozygous loss-of-function mutations in *PLN1* gene have been identified in FPLD patients, causing partial lipodystrophy and severe insulin resistance (91, 92). Functional characterization of three mutations in cellular experiments demonstrated that mutated perilipin-1 fails to repress basal lipolysis and prevents therefore from lipid accumulation (91–93). There are no knock-in mice for any of these mutations but PLN1-deficient mice have been generated. The initial publications characterized the effect of PLN1 deficiency under a mixed C57Bl6J/129 background (94). The WAT collected from *Pln1^-/-^* mice displays 70% mass reduction, small and abnormal LD and consistently with perilipin-1 function, elevated basal lipolysis and reduced adrenergic stimulated lipolysis (94). *Pln1^+/-^* mice tend to have a reduced WAT mass, which is however not significantly different from their wild-type littermates. When Perilipin-1 deficiency is produced under a pure C57/Bl6 background, the mice similarly display partial lipodystrophy but a more marked insulin resistance along with strong macrophage inflammation in WAT is observed (95). No report examined the phenotype of *Pln1^+/-^* under C57/Bl6 background. In summary, in humans, heterozygous frameshift mutations in perilipin-1 cause FPLD, a phenotype recapitulated in mice following homozygous perilipin deficiency.

### Hormone-Sensitive Lipase

In humans, bi-allelic null mutations in the *LIPE* gene, encoding the hormone-sensitive lipase (HSL), is associated with a complex AT phenotype including fat redistribution, multiple symmetric lipomatosis (excess fat accumulation) and partial lipodystrophy (96–98). At the metabolic level, the patients display different ranges of metabolic complications such as dyslipidemia, hepatic steatosis and systemic insulin resistance. In most cases, the consequence of HSL deficiency induces a late-onset disease with an age of diagnosis ranging from 23 to 76 years old. This complex phenotype could be attributed to the central role played by HSL in fat mobilization or lipolysis. Numerous studies on HSL-deficient mice are useful to further understand the pathophysiology involved.

Total HSL-deficiency strongly alters WAT properties resulting in blunted catecholamine stimulated lipolysis (99), heterogenous size of adipocytes (ranging from hypertrophic to abnormally small fat cells), DAG accumulation (100) and low adiponectin expression (101). The massive DAG accumulation likely contributes to the AT phenotype although this has not been formally demonstrated. In addition, AT stems cells isolated from a lipodystrophic patient carrying a bi-allelic *LIPE* null variant display impaired adipogenesis *in vitro *(97). However, it remains unclear how the clinical features can range from lipomatosis to partial lipodystrophy. Transgenic expression of human HSL gene restored normal adipose tissue mass, histology and circulating leptin levels (102). The glucose homeostasis status generated quite a lot of discussion and whereas some reports suggested that HSL deficiency might increase insulin sensitivity (101), others supported an impairment in glycemic control (103, 104), including a default in insulin secretion (105). Interestingly, whereas one study has shown that under short a HFD exposure (3 weeks), HSL deficiency protects the animals from the adverse effect of HFD (106); in an ob/ob background, HSL deficiency worsens the glucose homeostasis dysfunction (107). A recent study helped us to reconciliate these different findings. At 3 months of age, the HSL-KO mice display normal body weight, normal AT, and improved insulin sensitivity as compared to control mice (108). By contrast, 8-months old animals display lower body weight, progressive lipodystrophy along with AT macrophage infiltration, liver steatosis and insulin resistance (108). Finally, using liver and adipocyte-specific deletion of HSL, they demonstrated that only adipocyte HSL-deficiency recapitulates the whole phenotype reported in the global KO mice (108). Taken together, these studies collectively reported that, according to the age and energy balance status, HSL-deficiency might be temporary protective, but appears deleterious with ageing and increased energetic supply. This is compatible with the late-onset of the disease in humans.

### CIDEC

A female patient presenting with partial lipodystrophy insulin resistance and diabetes was found to be homozygous for a mutation (E186X) that leads to a premature truncation in the LD protein, cell death-inducing DFFA-like effector C (CIDEC aka FSP27) (109). Histological analysis of her AT revealed the presence of many multilocular adipocytes within the subcutaneous WAT depot. In cell experiments, the truncated protein does not surround the LD and fails to increase LD size, at the opposite of the WT protein (109). However, this mutation is not a loss-of-function and the protein still carry the CIDE-N domain. No mouse model of E186X-CIDEC has been generated, but CIDEC-KO mice are available. Interestingly, the white adipocytes of CIDEC-KO mice display multilocular LD phenotype, with increased mitochondria number and elevated FA oxydation (110). This adipocyte morphology resembles the one described in the lipodystrophic patient. However, under chow diet, CIDEC deficiency limits gain weight from 16 weeks and decreases random fed glycemia as well as it improves glucose tolerance. Under HFD, CIDEC-deficiency prevents weight gain and the appearance of the metabolic complications associated with obesity such as glucose intolerance and insulin resistance (110). The similar protecting effect was reported when CIDEC-deficient mice were crossed with ob/ob mice: the animals were leaner and have improved glucose homoeostasis (111). Similarly, to CIDEC KO mice, adipocyte-specific CIDEC deletion using aP2-CRE mice leads to a similar multilocular adipocyte phenotype. HFD-feeding of adipocyte-specific CIDEC deletion prevents from body weight gain but leads to insulin resistance, elevated plasma TG and FFA, and liver steatosis (112). CIDEC expression is 10-fold increase in the liver of these animals and liver-specific CIDEC overexpression has been shown to induce liver steatosis (113). In order to reconciliate these data, further studies on adipocyte versus liver CIDEC contribution are needed. Of note, the adipocyte specificity and the efficiency of aP2 CRE has been challenged (114) and might explain in part this unexpected phenotype.

### AKT2

One single family has been identified with partial lipodystrophy and severe insulin resistance that is due to a missense mutation in the *AKT2* gene encoding the key insulin signalling ser/thr kinase. This R274H substitution shows an autosomal dominant transmission and exerts a dominant negative-effect on wild-type AKT2, compromising insulin signalling in hepatocytes and adipocyte cell lines (115). AKT2-deficient mice display severe systemic and muscle insulin resistance (116, 117) and progressive lipodystrophy affecting all WAT depots (117). Interestingly, adipocyte-specific deletion of AKT2 is sufficient to recapitulate severe lipodystrophy, liver steatosis and hyperglycemia and hyperinsulinemia despite normal glucose tolerance (118). The severe AT loss in this model is consistent with the phenotype reported in Adipo-ind-IRKO (76) (cf 2.5) supporting a central role of adipocyte insulin signalling in controlling adipocyte good health. On the other hand, the fact that adipocyte-specific deletion of AKT2 does not impair glucose tolerance whereas insulin receptor deletion does, and given that lipodystrophy is similarly severe in both mice, is quite unexpected. Both models display increased beta-cell mass, but as the insulin receptor deletion is inducible and leads to rapid AT loss and lipid spill-over, it is possible to speculate that an adaptative response in constitutive AKT2-deficient mice induces a stronger hyperinsulinemia that compensates for insulin resistance and allows normal GTT. However, in refed conditions, AT deletion of AKT2 leads to hyperglycemia. In conclusion, both models demonstrate that adipocyte insulin signalling control AT properties and systemic glucose homeostasis. Regarding AKT2, whereas there are so far no mouse model harbouring the R274H mutation described to lead to human partial lipodystrophy and severe insulin resistance, adipocyte-specific AKT2 deficiency recapitulates lipodystrophy associated with impaired glucose homoeostasis.

## It Is Not That Easy to Recapitulate FPLD

Most of the mice models of FPLD, generated either by loss-of-function mutations or knock-in of FPLD genes, display healthy leanness, under chow diet. At the opposite, under extremely obese background as for ob/ob mice, limited AT expansion leads to severe metabolic complications. Under HFD conditions, the results are more variable, since short HFD-fed mice display healthy resistance to weight gain whereas longer HFD exposure unmasks insulin resistance, glucose intolerance and liver steatosis. To this extent, the recent longitudinal study reporting a time course in HSL KO mice is very informative and supports the idea that the metabolic consequences are dependent on the storage imbalance. Thus, mice can cope with lower adipose storage capacity when facing moderate energy excess whereas, in conditions of severe hyperphagia (ob/ob) or chronic HFD, the limit of expansion of AT is reached leading to subsequent metabolic complications. Moreover, the analyses of rodent models for FPLD tend to suggest that mice can cope more easily with limited expansion of AT than humans. The example of P465L-PPARγ mice is striking: those mice develop insulin metabolic complications only under very high energy intake (ob/ob background), whereas P467L patients are diabetic. One important element that should be considered here is the heterogeneity of the different fat pads behaviour. Indeed, in humans, FPLD is characterized by loss of limbs WAT but the mass of the other fat pads, including truncal WAT is either unchanged or increased. The reason for this difference in fat pad behaviour remains unknown. In most of the rodent models discussed here, the AT loss is similar between the different WAT depots, which is already a strong difference with humans. Furthermore, in R482Q-LMNA and P465L-PARγ, the inguinal fat pad is less severely affected. This might explain the difference in the severity of the phenotypes. It is now well established that rodent and human fat pads display strong differences. The rodent gonadal WAT which has been used in most of the studies presented here as visceral AT, is very small in human where visceral fat is more represented by mesenteric and omental WAT (119). Mice inguinal WAT is largely used to study subcutaneous WAT but human subcutaneous fat pads are divided into upper and lower subcutaneous WAT questioning the relevance of using inguinal WAT as a mirror depot of human lower subcutaneous WAT (120). All these key questions call for caution in interpretating rodent experiments with respect to WAT heterogeneity. The recent single cell/nucleus studies in human and mice should bring new valuable information regarding the diversity of progenitors and mature adipocytes in the different fat pads (121, 122).

## Conclusion and Perspective

Collectively, generalized lipodystrophy mouse models strongly demonstrate that the lack of AT storage leads to ectopic fat deposition, triggering the development of diabetes mellitus. Importantly, NAFLD, diabetic cardiomyopathy, kidney disease and beta-cell dysfunction have been largely studied in BSCL rodent models and further demonstrate the implication of AT failure in the development of cardiometabolic diseases. In addition, the use of adipose-specific tools strongly demonstrates that, most of the time, the metabolic abnormalities are linked to the specific deficiency of the BSCL gene in the adipocytes and that adipocyte implementation is sufficient to restore normal insulin sensitivity, glucose tolerance and to correct liver steatosis. It is therefore obvious that complete or major lack of AT is deleterious for metabolic health and that rodent models are very useful. However, as discussed earlier, it is more difficult to predict the consequences of limited adipose expansion capacity. Indeed, in FPDL models the frontier between leanness and lipoatrophic diabetes is not always obvious and seems to depend on the level of positive energy balance. Based on the data reported here, we would propose that ob/ob background is probably the option to unmask adipose failure phenotype for FPLD genes. The phenotype of FPLD mice also raises the question of what is the rodent fat pad that represents the best omental AT, i.e., typical visceral fat in human, and similarly for gluteofemoral, i.e., subcutaneous WAT in human. In conclusion, rodent models are useful but not perfect tools and *in vitro* studies are crucial to decipher the individual function of the genes involved in inherited lipodystrophies.

## Authors Contributions

SL, JM, and XP wrote the manuscript. All authors contributed to the article and approved the submitted version.

## Funding

This study received a grant from Fédération Française des Diabétiques (FFRD) that includes funding from Abbott, AstraZeneca, Eli Lilly, Merck Sharp & Dohme (MSD) and Novo Nordisk. The funder was not involved in the study design, collection, analysis, interpretation of data, the writing of this article or the decision to submit it for publication.

## Conflict of Interest

The authors declare that the research was conducted in the absence of any commercial or financial relationships that could be construed as a potential conflict of interest.

## Publisher’s Note

All claims expressed in this article are solely those of the authors and do not necessarily represent those of their affiliated organizations, or those of the publisher, the editors and the reviewers. Any product that may be evaluated in this article, or claim that may be made by its manufacturer, is not guaranteed or endorsed by the publisher.
